# Electronic Nose for Quality Control of Colombian Coffee through the Detection of Defects in “Cup Tests”

**DOI:** 10.3390/s100100036

**Published:** 2009-12-24

**Authors:** Juan Rodríguez, Cristhian Durán, Adriana Reyes

**Affiliations:** Department of Electronic Engineering, University of Pamplona, Pamplona, 057, 75, Colombia; E-Mails: ju4n@unipamplona.edu.co (J.R.); areyes@unipamplona.edu.co (A.R.)

**Keywords:** quality control, Colombian coffee, chemical sensors, data acquisition, data processing

## Abstract

Electronic noses (ENs), are used for many applications, but we must emphasize the importance of their application to foodstuffs like coffee. This paper presents a research study about the analysis of Colombian coffee samples for the detection and classification of defects (*i.e.*, using “Cup Tests”), which was conducted at the Almacafé quality control laboratory in Cúcuta, Colombia. The results obtained show that the application of an electronic nose called “A-NOSE”, may be used in the coffee industry for the cupping tests. The results show that e-nose technology can be a useful tool for quality control to evaluate the excellence of the Colombian coffee produced by National Federation of Coffee Growers.

## 1. Introduction

The coffee is one of the most important beverages in the world. Depending on the quality control needs of coffee, analysts have developed different types of analysis of green and roasted coffee, in order to obtain a standard product for the market.

The coffee quality is influenced by many factors, depending on the amount of time that the coffee is stored in the Almacafé warehouses; there are some protocols for determining quality and the purchase price from the farms. The good condition, appearance and proper size of the coffee grains is important when assessing a sample, but further tests are done in the cup or by tasters.

The cup tests are performed by expert tasters and some are in the training process. This way of conducting quality control is very subjective, depends on the skill of the taster and the training that he/she has. Moreover, not all the tasters are able to find every defect in a cup of coffee. This problem sometimes causes loss of money, time and failure in the delivery of the export. This is due to a lack of standardization of cupping tests, which could be solved by making a classification of coffee grains based on a more reliable organoleptic analysis [*i.e.*, an electronic nose (EN)] to identify defects in cups as the tasters do, but based on patterns of training so that the analysis and results are not affected by external factors.

Green and roasted beans contain an appreciable amount of aliphatic hydrocarbons, probably derived from the oxidation of green bean lipids during storage or transport prior to roasting [1]. The combination of gas chromatography and mass spectroscopy (GC–MS) is by far the most popular technique for the identification of volatile compounds in foods and beverages [2–4], still used to date in coffee quality control laboratories but they are very expensive and laborious.

Applications of electronic noses in the coffee sector have included analysis of different types of coffee [5], evaluation of the quality of Italian espresso coffee in order to determine the best time for packaging [6], identification of various brands and mixtures [7], detection of aromas in the coffee powder [8], classification of samples with different roasting levels and commercial coffee blends [9,10]. Other studies were made to classify different monocultivar coffees and commercial coffee blends [11], to evaluate coffee ripening [12] and also to validate a commercial electronic nose using mixtures of three coffees [13], but none have reported a study on the quality control of Colombian coffee. In this paper we describe an EN applied to detect defects in Colombian coffee cup tests [14].

In this study, almond Arabica type coffee was used for showing the results of discrimination levels, using PCA (Principal Component Analysis), and the validation process, using Artificial Neural Networks (ANN). Different coffees with and without defects were also analyzed in the eventual roasting and cup tests.

## 2. Experimental

### 2.1. General Configuration

In this experiment we used an electronic nose called the “A-NOSE”, developed at the University of Pamplona (Colombia) [15]. This equipment is composed primarily of four parts with different functions.

The first is to conform the adequacy of the gas mixture and sampling. This is done through the concentration chamber, which is destined to store the samples of the product (solid or liquid) that is being analyzed; this chamber has the option of warming the samples. The dynamics of the system includes two three-way valves and an air pump, which are used in the three stages of process: concentration, measurement and purge; they are controlled by means of a PIC16F877A, which simultaneously drives a screen alphanumeric LCD (Liquid Crystal Display) that visualizes messages corresponding to every stage. The PIC (Programmable Integrated Circuit) also takes charge of controlling the rest of the system that includes a fan to refresh the interior of the device and the heating element of the chamber of concentration, the synchronization between the PIC and the data acquisition card DAQ (Data Acquisition) USB 6009 is done by three digital information lines.

The second part of the device is developed by several gas sensors that detect the volatile compounds, in this case is a matrix of eight metal-oxide gas sensors manufactured by Figaro Inc (TGS sensors) and FIS Inc. (SP sensors). Gas sensors are widely used to assemble arrays for odour measurement. The gas is sensed by its effect on the electrical resistance of the tin-dioxide semiconductor, resulting from changes in conductivity brought about by combustion reactions ocurring with lattice oxygen species on the surface of the tin-dioxide particles [16]. Table 1 shows the gas sensors used; these types of sensors were chosen because of their high sensitivity to organic, natural, pollutant, and combustible gases.

The third part is composed by the electronics’ control and data acquisition systems, an important part for controlling the sensor array, system dynamics and the data acquisition process. As mentioned before, the data acquisition is made through a DAQ USB6009 device that plugs into the USB port. The device uses eight analog input channels to acquire signals from eight gas sensors and uses three channels configured as digital inputs and outputs for providing communication with the microcontroller.

The fourth and last part of the equipment is the feature extraction software that obtain the main features or “mark” of each scent, and applys pattern recognition methods (e.g., Artificial Neural Networks and Principal Components Analysis)

Initially some measurements were made with green coffee beans. For this procedure we took some samples of dry parchment coffee, which was stripped of the husk by a threshing machine (you obtain in this way the green coffee beans that are ready to export or for industrial processing); after this process samples (approximately 5 g) were taken and deposited in the concentration chamber of the equipment, to perform the measurement process, but the response of gas sensors and results were not the best because when coffee beans are green, the scent is not very strong and it is difficult to detect with the electronic nose.

Subsequently we tested with ground coffee, for this procedure we took some samples of green coffee beans, which underwent a roasting procedure in a specialized machine in laboratory; once the coffee reached the proper degree of roasting it was placed in a machine that performed the grinding of coffee to obtain the coffee grounds (ready to drink). To carry out tests with ground coffee it was determined that addition of water was necessary because the system has an air pump that is used to drag the volatiles of the samples to the sensors chamber, and in this case, the air pump dispersed particles of the ground coffee sample throughout the whole equipment, which is undesirable. In addition it was modeled after conducting coffee cup tests (coffee tasting), where the ground coffee has hot water added at a temperature about 140 °F, which facilitates the measurement process, since coffee generates more volatiles.

The first test measurements were used to determine the conditions for the measurement process. Later a set of measurements were carried out with some types of coffee: “Excelso UGQ2, “Excelso Europa”, “Pasillas”, and coffee with defects (e.g., black, white, vinegar). The defects detected in coffee cupping, are often caused by a defect in the coffee beans; these defects are identified visually, but the degree of impact depends on the percentage of defects found in a given sample. Once the measurements were obtained, the PCA technique was applied and a feed-forward backpropagation neural network (*i.e.*, MLP) was used as a data processing technique.

Principal Component Analysis (PCA) is an effective unsupervised linear method to project data from several sensors to a two-dimensional plane. Is a linear method that has been shown to be effective for discriminating the response of an EN to simple and complex odours [17]. The neural network model used in this study was a multilayer perceptron (MLP) that learns by using an algorithm called backpropagation with an adaptive learning rate [18]. Therefore, in this application a MLP (Multilayer Perceptron) network with ten hidden and one output neurons was applied to achieve a success rate in classification a cross-validation technique called “leave one out” of order one, was implemented to estimate the success rate in classification. A leave-one-out estimates the performance of the network in the classification of coffee samples. This interactive validation approach generates N evaluation procedures (1 for each measurement). For each iteration, a different measurement is left out, while the remaining measurement (the one not used for training) is then projected onto the neural model and classified using the already trained network. This is repeated N times (one for each measurement) so that the final result is the average success of entire iterative process. The data pre-processing and processing were done with algorithms written in Matlab 7.5 having added a Graphical User Interface (GUI).

### 2.2. Parameters Used in the Measurement Processes

The coffee beans analyzed were roasted and ground and 2 g of ground coffee were used for each measurement. Eight g of water were added to facilitate the extraction of volatiles (for a total weight of 10 g for the ground coffee and water). The water temperture was 140 °F and room ambient temperature was 75 °F. The volume of the concentration chamber was approximately at 0.0034 m^3^.

#### Measurement Times:

**Figure 2.**
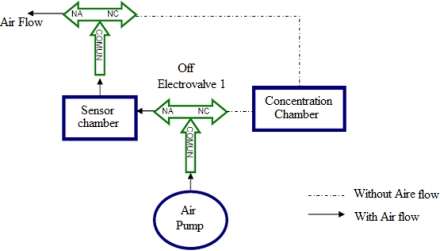
Diagram of air flow in the concentration and purge stages.

**Figure 3.**
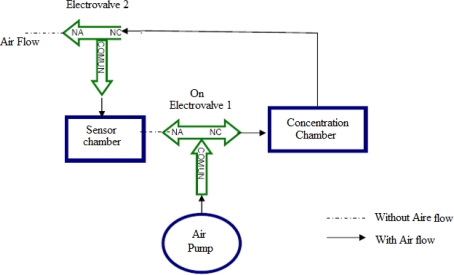
Diagram of air flow in the stage of Measure.

1. *Concentration*: This takes 5 minutes. It is the first stage of the measurement process, and it aims to concentrate the volatiles of a sample (e.g., coffee) in the concentration chamber, which is accomplished by disabling two electro-valves arranged for one of two routes into and another at the exit, to isolate the interior of the concentration chamber with the external environment. At this stage, it also activates the air pump to direct the flow toward the exit, passing by the sensor chamber, in order to clean and remove waste volatiles remaining from other measurements. See Figure 2.

2. *Measurement:* This takes 5 minutes. At this stage of the measurement process, we acquire the signals from the gas sensors therefore it must activate the two electro-valves and air pump simultaneously to direct the flow of air from the pump to the concentration chamber, dragging the volatiles towards the sensor chamber. See Figure 3.

3. *Purge*: This takes 5 minutes. It is the last stage of the measuring process, the goal is to clean and remove volatile residues of the previous measure, the two electro-valves were turned off and the air pump was activated. In this case, the concentration chamber can be opened manually to remove the concentrated sample, helping to eliminate the volatiles concentrated in it to begin a new measurement. The process can be seen in Figure 2, which is a similar process but without sample concentration. The time to each measure with coffee samples was 15 min.

### 2.3. Equipment Configuration

**Figure 4.**
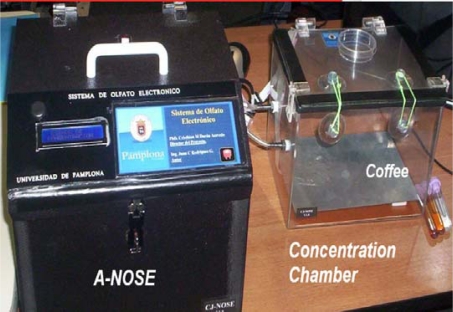
“A-NOSE” Electronic Nose System.

Figure 4 illustrates the final system configuration with the different subsystems. In order to obtain repetitive results, the whole measurement process had to be automated. In fact, the automation of the equipment was one of the initial goals that had to be proven if the system is ever to become commercially available. A PC was in charge of the measuring process, controlling each subsystem (e.g., concentration chamber, sensor chamber, *etc.*).

The first analysis was made with two types of good coffee. The first was “Excelso UGQ”; this is an export coffee, in which one can find up to 72 defective grains in a sample of 500 g. The other kind of good coffee was manually selected to provide “Healthy Coffee” Pasillas of coffee beans.

Data preprocessing was applied with the static parameter, which is obtained from the variation of maximum conductance (Gmax) and minimum conductance (Gmin) and the “mean-centring method was used to the data matrix before the PCA was performed.

The second analysis was conducted to compare the different samples of Excelso coffee, three samples of coffee with cup defects and three samples of totally flawed coffee.

The samples of good coffee were taken from “Excelso UGQ”, “Healthy Coffee” and “Excelso Europe”. The first two groups correspond to the same measurements used in the first analysis, while the “Excelso Europe” is a type of export coffee which is preferred by customers. The coffee samples with “cup defects” were composed of “reposed coffee”, “fermented coffee” and “coffee with chemicals”. The coffee with chemicals was obtained from some Pasillas, which were previously used in cupping tests. The completely flawed coffee samples were obtained by manually selecting the grains of the Pasillas white, black and vinegar coffee beans.

## 3. Results and Discussion

### 3.1. First Analysis

**Figure 5.**
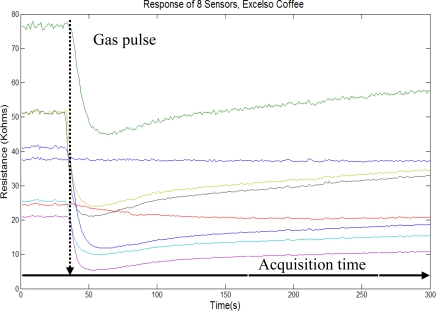
Sensor array response for Excelso coffee.

**Figure 6.**
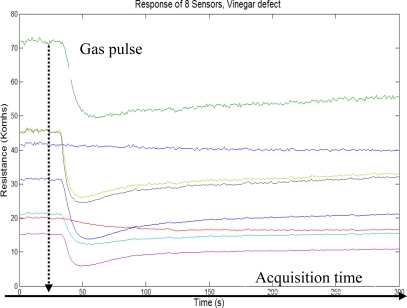
Sensor array response for vinegar coffee.

One of the analysis which was carried out included the comparison of some types of Excelso coffee (*i.e.*, coffee of excellent export quality), Pasillas coffee (*i.e.*, coffee with many defects, which do not comply with the export standards). Figures 5 and 6 show the typical sensor array responses with roast coffee (*i.e.*, Excelso and Pasillas with vinegar). Each figure contains the indication of gas probe pulse (“Dashed line”) and acquisition time (“Solid line”) of five minutes.

These two types of coffee were compared with two samples of Pasillas, which were tested and detected in cup tests. According to the taster’s opinion, one of these coffee samples had “reposed coffee or restful coffee” notes and the other “fermented coffee”. Of these two defects the most common is reposed coffee, caused by prolonged storage or poor storage conditions. The fermentation is presented by some of the following factors: lack of water during the development of the plants, delays between the collection and/or processing and others.

**Figure 7.**
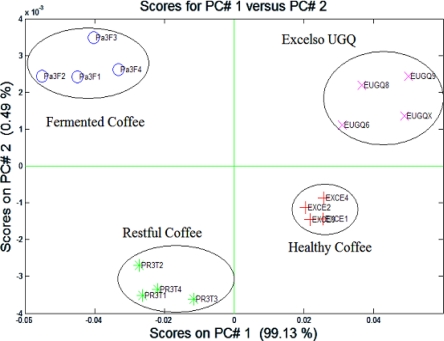
PCA graph with different types of cluster (coffee with defects and without defects).

Figure 7 illustrates the results of PCA which were applied to the measurements mentioned above. The samples of good coffee, which were “healthy coffee” and “Excelso UGQ”, are located on the right side of the graph. The coffee samples with defects in the cup, such as reposed coffee and fermented coffee, are located on the left side. Each group is composed of four samples, for a total of 16 measures (as it was not harvest season, there was difficulty in obtaining a larger set of samples).

**Figure 8.**
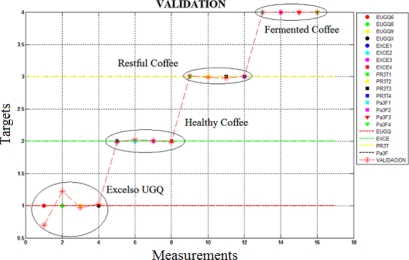
Validation of the measures in the first analysis with the MLP Neural network.

The figure depicted the first two principal components represented in 99.62% of the variance in the data set. Additional to the PCA a feed-forward backpropagation neural network was applied and the validation was obtained with the “leave-one-out” method, as can be seen in Figure 8, where a classification rate of 100% was achieved.

### 3.2. Second Analysis

**Figure 9.**
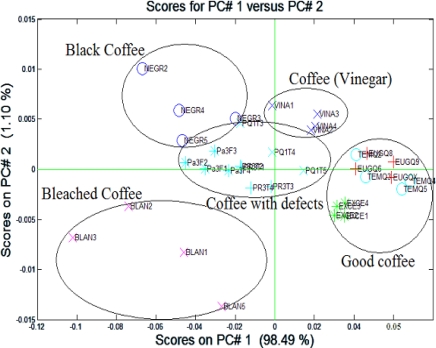
PCA graph with more types of cluster.

Figure 9 shows the two first PC’s account for more that 99% of the variance in the data. The samples of good coffee, such as, “healthy coffee”, “Excelso UGQ” and “Excelso Europe”, are located on the right side of the figure. The coffee samples with coffee with defects are “repose coffee”, “fermented coffee” and “coffee with chemicals”. These are located in the center. The completely flawed coffee samples are skirting the coffee with defects in cup. The flawed coffee is related to the coffee with defects in the cup. Each group was composed of four measurements, for a total of 35 measures.

**Figure 10.**
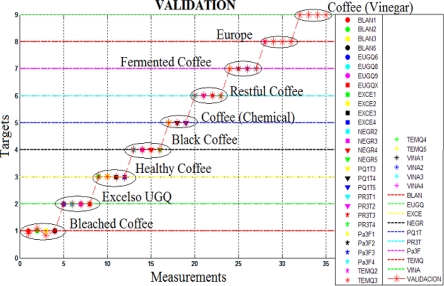
Validation of the measures in the second analysis with the neural network.

As shown in Figure 10, the feed-forward backpropagation neural network obtained a classification success rate in nine categories, reaching a 92.5% using the leave-one-out approach.

## 4. Conclusions

The sensitivity obtained by the gas sensors was good, and the selectivity shown by the equipment was adequate. In addition, data processing with PCA and neural networks, was successful. An electronic nose can be used for quality control in the coffee sector, since it can classify different samples of Excelso coffee, coffee with defects in the cup and others. The green coffee samples could not be classified by the equipment due to low concentrations or emissions of volatiles from the coffee beans. It is important to make more measurements to find the efficiency of the equipment, in particular, on-line application in the coffee sector.

## Figures and Tables

**Figure 1.**
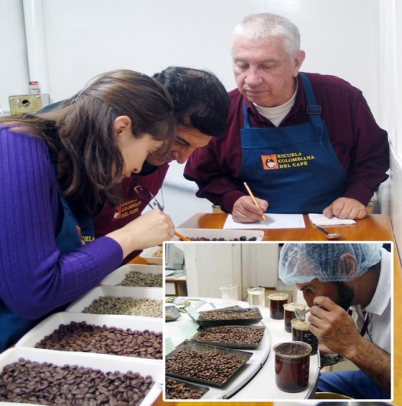
Professional Colombian coffee tasters school.

**Table 1.** Sensor array description.

**Amount**	**Sensor**	**Application**
1	SP-12A	Flammable Gases
1	SP-31	Organic Solvents
1	TGS-813	Combustible gas
1	TGS-842	Methane, natural gas
1	SP-AQ3	Air Quality Control
1	TGS-823	Combustible Gases
1	ST-31	Organic Solvents
1	TGS-800	Air Quality, Smoke, Benzene
